# Alien Hand Syndrome Following Pontine Hemorrhage: A Case Report of Rare Mixed Phenomenology

**DOI:** 10.3390/reports9030233

**Published:** 2026-07-21

**Authors:** Ülkü Figen Demir, Fatmanur Karakuş Dilbaz, Nur Banu Memur

**Affiliations:** 1 Department of Neurology, Faculty of Medicine, Istanbul Yeni Yüzyıl University, Istanbul 34010, Turkey; fatmanur.karakus@yeniyuzyil.edu.tr; 2 Department of Internal Medicine, Faculty of Medicine, Istanbul Medeniyet University, Istanbul 34700, Turkey; nurbanumemur44@gmail.com

**Keywords:** alien hand syndrome, alien limb phenomenon, pontine hemorrhage, pons, proprioception, sensory ataxia, intermanual conflict, case report

## Abstract

**Background and Clinical Significance**: Alien hand syndrome (AHS) is a rare disorder of agency and complex motor control characterized by involuntary, apparently purposeful limb movements experienced as outside voluntary control. Pontine hemorrhage is an uncommon substrate, and its manifestations may overlap with sensory ataxia and other post-stroke movement disorders. **Case Presentation**: An 86-year-old right-handed man developed right-sided alien hand phenomena after a left pontine hemorrhage. Examination showed dysarthria, limited left gaze, diplopia, preserved muscle strength, marked right-sided proprioceptive impairment, a thalamic-hand-like posture, impaired spatial control, involuntary levitation, intermanual conflict, and purposeful-appearing rubbing movements when distracted. The diagnosis was based on loss of agency and autonomous limb behavior that could not be explained by sensory ataxia alone. Serial CT demonstrated an interval reduction in the size of the pontine hemorrhage; a representative thalamic level CT showed no evident thalamic hemorrhage or gross structural lesion, although a small CT occult ischemic lesion could not be excluded. Repeat MRI was not completed because of severe claustrophobia and anesthesia risk. EEG, formal neuropsychological testing, and standardized functional scales were unavailable. The NIHSS, assessed 15 days after admission to our hospital, was 6 points. No specific pharmacological treatment was initiated. Cognitive-behavioral rehabilitation was recommended, but transportation difficulties prevented regular attendance. Approximately three months after discharge, physician relatives reported resolution of abnormal movements and improved independent gait; no formal post-discharge examination was performed. **Conclusions**: Pontine hemorrhage may rarely be associated with mixed AHS phenomenology. Disruption of ascending proprioceptive and sensorimotor pathways is plausible, but the absence of advanced imaging and neurophysiological assessment precludes definitive anatomical or causal conclusions.

## 1. Introduction and Clinical Significance

Alien hand syndrome (AHS) is a rare disorder of agency and complex motor control in which a limb performs involuntary, often apparently purposeful movements that the patient experiences as being outside voluntary control [1,2,3,4].

Goldstein provided one of the earliest classic descriptions in 1908, and Brion and Jedynak later introduced the concept of the foreign hand in patients with callosal lesions [5,6]. AHS is now commonly described in frontal, callosal, posterior, or mixed forms [2,3,7,8,9,10,11,12,13]. Frontal manifestations include grasping, groping, and compulsive object use; callosal disease is characterized particularly by intermanual conflict; and posterior forms may include levitation, sensory ataxia, visuospatial disturbance, and altered limb ownership [2,3,8,9,10,11,12].

Reliable population-based estimates of AHS prevalence and misdiagnosis are not available because the syndrome is rare, terminology is heterogeneous, and the literature is dominated by individual case reports and small series [2,3,4]. Failure to recognize the syndrome may lead to psychiatric misattribution, unnecessary treatment for other movement disorders or epilepsy, delayed neuroimaging, and delayed rehabilitation. A search of the current World Health Organization ICD-10 and ICD-11 online browsers does not identify AHS as a standalone named category [14,15]. The underlying neurological lesion is therefore generally documented etiologically, while AHS is recorded clinically. The International Classification of Functioning, Disability and Health may complement etiological coding by describing impairments in voluntary motor control, proprioception, hand use, mobility, and activities of daily living [16].

The diagnosis is clinical and depends on careful characterization of loss of agency or ownership, autonomous goal-directed activity, intermanual conflict, grasping or groping, limb levitation, and associated sensory or visuospatial deficits. Severe proprioceptive loss, sensory ataxia, apraxia, focal seizures, chorea, dystonia, functional movement disorders, and medication-related movements may produce partially overlapping findings and should be considered in the differential diagnosis [2,3,4].

Most reported cases involve the corpus callosum, medial frontal region, parietal or occipital cortex, or thalamus. Pontine involvement is exceptional, although several pontine hemorrhage cases have been reported [17,18,19]. The purpose of this report is to present the clinical phenomenology, diagnostic reasoning, management, and longitudinal outcome of an 86-year-old man with right-sided alien hand phenomena after a left pontine hemorrhage. The principal contribution is the coexistence of posterior-type levitation and proprioceptive impairment, callosal-type competitive reaching, and frontal-like involuntary manipulation in a dominant hand after an infratentorial lesion.

## 2. Case Presentation

This case report was prepared in accordance with the CARE reporting guideline [20].

### 2.1. Patient Information and Initial Presentation

An 86-year-old right-handed retired agricultural engineer was alone in his summer house when he experienced a sudden sensation of heat in his head, described as if hot water had been poured over him. He attempted to seek help from his neighbors, then lost consciousness with fecal incontinence. After regaining consciousness, he again lost consciousness while attempting to go to the bathroom to clean himself, fell forward, and sustained a wound over the left upper eyebrow. He was taken to hospital, where non-contrast cranial computed tomography (CT) demonstrated a left pontine hemorrhage (Figure 1). A representative axial CT image through the thalami showed no evident thalamic hemorrhage or other gross structural lesion (Figure 2). According to the discharge summary provided by the patient, diffusion-weighted magnetic resonance imaging performed during the initial hospitalization revealed no evidence of acute ischemia. He was monitored in an intensive care unit for two weeks and was then transferred to our hospital for neurological management.

His medical history included controlled type 2 diabetes mellitus, controlled hypertension, a cardiac stent, and antiplatelet therapy. He lived alone, had no previous cognitive complaints, and had no documented family history of cognitive impairment. Before the event, he was independent in activities of daily living and maintained an active lifestyle that included regular swimming and walking. His body mass index was within the normal range. He had no history of alcohol or tobacco use.

### 2.2. Timeline

The clinical course is summarized in Table 1.

### 2.3. Neurological Examination

On arrival at our hospital, the patient was conscious, oriented, and cooperative. Speech was dysarthric and comprehension was preserved. Left lateral gaze was limited and he reported diplopia. Muscle strength was globally preserved. No major superficial sensory deficit was identified, whereas deep sensory function and proprioceptive awareness were prominently impaired in the right upper and lower extremities. He also described tightness in the right forearm; however, neither rigidity nor cogwheel phenomenon was detected on examination. Deep tendon reflexes were hypoactive in all extremities. Plantar responses were mute bilaterally.

A posture resembling a thalamic hand was observed on the right, with wrist flexion and slight hyperextension at the metacarpophalangeal joints, becoming more evident when he extended the right hand forward. He repeatedly stated that the right hand moved on its own and rose involuntarily. He described intended movement as being transmitted incompletely from the shoulder and upper arm to the hand, as if the arm consisted of three separate parts loosely connected like a puppet. A forced grasp response was not documented.

Spatial control of the right hand was impaired with respect to speed and target direction. When he attempted to grasp an object with the left hand, the right hand competitively reached for the same object, consistent with intermanual conflict. Finger-to-nose testing was normal on the left; on the right, the index finger deviated toward the left ear rather than reaching the nose. He was unable to hold his ear or reliably grasp a targeted object with the right hand. At rest, particularly when attention was directed elsewhere, apparently purposeful right-hand movements such as rubbing the bedsheet were observed. Similar but milder spatial control difficulty was present in the right lower extremity. Sitting balance was impaired, with a tendency to fall backward and to the right, and he could not walk without support. The abnormal hand behavior was observed while he was fully conscious and was not described as a brief, stereotyped, paroxysmal event.

### 2.4. Diagnostic Assessment

Routine laboratory investigations did not identify an alternative metabolic explanation for the abnormal movements. Given the history of hypertension and diabetes mellitus, the pontine hemorrhage was attributed clinically to small-vessel disease. Serial cranial CT demonstrated a reduction in hemorrhage size. A representative axial CT image through the thalami showed no evident thalamic hemorrhage or other gross structural lesion (Figure 2). According to the discharge summary from the referring center, diffusion-weighted MRI performed during the acute phase showed no evidence of acute infarction. After admission to our hospital, repeat diffusion-weighted MRI was planned; however, the examination could not be completed because the patient developed severe claustrophobia during the initial attempt. Considering the risks of performing MRI under anesthesia in this 86-year-old patient, and in accordance with the wishes of his physician relatives, repeat MRI was not pursued. No alternative advanced neuroimaging modality was performed. Formal neuropsychological testing, standardized apraxia or neglect assessment, electroencephalography, and video-based movement analysis were not performed. The National Institutes of Health Stroke Scale (NIHSS), assessed 15 days after admission to our hospital, was 6 points.

### 2.5. Differential Diagnosis and Diagnostic Reasoning

Marked proprioceptive impairment and sensory ataxia were important components of the presentation and probably contributed to dysmetria and impaired target reaching. However, sensory ataxia alone did not fully explain the patient’s explicit loss of agency, involuntary limb levitation, competitive goal-directed reaching with the opposite hand, and apparently purposeful manipulation of the bedsheet. The overall phenomenology was therefore considered most consistent with AHS with mixed features. The principal alternative diagnoses and the available reasoning are summarized in Table 2.

### 2.6. Management and Follow-Up

No specific pharmacological treatment for AHS was initiated because the involuntary movements were not considered sufficiently severe to justify empiric medication. Cognitive-behavioral rehabilitation was recommended, but the patient was unable to participate because transportation difficulties prevented regular outpatient attendance. Physical therapy and rehabilitation were advised. Approximately three months after discharge, according to information obtained from the patient’s physician relatives, the abnormal movements were no longer observed and independent gait had improved. No formal neurological examination was performed at this time. Approximately two years after the hemorrhage, according to information obtained from the patient’s physician relatives, no evident cognitive decline was reported; this later information was also not supported by formal neurological or neuropsychological testing.

The available follow-up and outcome information is summarized in Table 3.

### 2.7. Focused Literature Search

To contextualize the rarity of pontine localization, a focused PubMed search was performed from database inception through 13 July 2026 using combinations of the terms “alien hand”, “alien limb”, “pons”, “pontine”, “brainstem”, and “hemorrhage”. This was a targeted, non-systematic search and did not include formal study selection or risk-of-bias assessment. At least three previous reports describing alien hand or closely related posterior alien limb phenomena after pontine hemorrhage were identified [17,18,19]. Accordingly, the present case is described as one of a small number of reported pontine cases rather than as the second case in the literature.

## 3. Discussion

AHS is clinically heterogeneous. Frontal variants are associated with grasping, groping and stimulus-bound object use; callosal variants are characterized especially by intermanual conflict; and posterior variants may present with levitation, sensory ataxia, visuospatial disorganization, and disturbed limb ownership [2,3,8,9,10,11,12]. Mixed phenotypes have also been reported [3,13]. The descriptive term “mixed” is appropriate in the present case because posterior-type levitation and proprioceptive impairment coexisted with competitive bimanual behavior and frontal-like manipulation.

The most important diagnostic challenge was distinguishing AHS from the direct consequences of pontine sensory pathway injury. Severe proprioceptive impairment and sensory ataxia plausibly accounted for dysmetria, inaccurate reaching, and part of the abnormal posture. Diagnostic confidence arose from the additional presence of an explicit loss of agency, repeated involuntary levitation, competitive reaching toward objects selected by the opposite hand, and apparently purposeful rubbing behavior. These features are not fully explained by sensory ataxia alone. Nevertheless, AHS has no universally validated diagnostic test, and the absence of standardized apraxia, neglect, agency, and movement assessments requires a cautious interpretation.

The focused literature search identified several relevant pontine reports. Rafiei and Chang described right sensory alien hand phenomenon after left pontine hemorrhage [17]. Mirza et al. reported levitation and enabling synkinesis after a right dorsal pontine tegmental hemorrhage [18]. Hosokawa et al. described suspected posterior AHS accompanied by a supernumerary phantom limb after pontine hemorrhage [19]. The present case adds a dominant-hand presentation with combined levitation, intermanual conflict, involuntary manipulation, and mild lower-limb involvement. These reports collectively indicate that pontine lesions may rarely be associated with alien limb phenomena, but they do not establish a single uniform pontine syndrome.

A possible explanation is that disruption of ascending proprioceptive pathways, including medial lemniscal and pontine tegmental networks, interferes with the integration of sensory feedback, body schema, and frontoparietal motor control. Altered sensory feedback could impair the comparison between intended and executed movement and thereby disturb the sense of agency. This interpretation is biologically plausible but remains hypothetical [17,18].

Evidence for the treatment of AHS is limited, and management is largely symptomatic. Reported options include visuospatial and behavioral strategies, keeping the affected hand actively occupied, cognitive techniques, clonazepam, amantadine, and botulinum toxin in selected patients [3,4]. Because no specific pharmacological treatment was administered, treatment-related adverse effects were not observed or assessable in this patient. In this case, cognitive-behavioral rehabilitation was recommended, but regular outpatient participation was not feasible because of transportation difficulties. This documented logistical barrier illustrates how access constraints may limit post-stroke rehabilitation participation in older adults. The subsequent improvement, reported by the patient’s physician relatives despite the absence of structured cognitive-behavioral rehabilitation, may reflect spontaneous post-stroke recovery and neuroplastic reorganization; however, a direct mechanistic inference cannot be drawn from a single case.

The follow-up time points should be interpreted separately. At approximately three months after discharge, according to information obtained from the patient’s physician relatives, the abnormal movements had resolved and independent gait had improved. At approximately two years after the hemorrhage, information obtained from the patient’s physician relatives indicated no evident cognitive decline. Neither time point included a formal neurological or neuropsychological examination. The transient nature of the clinical manifestations and the absence of any cognitive decline or other signs suggestive of dementia during the two-year follow-up were considered to argue against corticobasal degeneration. The close temporal relationship to the hemorrhage, the focal ocular motor and sensory findings, and subsequent improvement support a vascular association. Clinicians should nevertheless consider neurodegenerative disease, focal seizures, medication effects, functional neurological disorder, apraxia, and other post-stroke movement disorders when evaluating similar presentations.

From a rehabilitation perspective, systematic documentation of episode frequency, task-specific limb control, gait, activities of daily living, and caregiver observations would strengthen future reports. Video documentation, standardized functional measures, and repeated bedside examination could help distinguish spontaneous recovery from treatment-associated improvement and could improve comparison across the small number of published cases.

### 3.1. Strengths and Limitations

The principal strength of this report is the detailed bedside description of a rare and clinically complex alien hand phenotype after pontine hemorrhage. Important limitations include the inability to obtain repeat MRI or alternative advanced neuroimaging; the absence of EEG, formal neuropsychological testing, standardized apraxia or neglect assessment, and video analysis; the lack of prospectively recorded functional scales; follow-up based on information obtained from the patient’s physician relatives rather than direct examination; and the inability to evaluate the effect of cognitive-behavioral rehabilitation because transportation difficulties prevented regular outpatient attendance. The focused literature search was not systematic. These limitations restrict anatomical localization, objective outcome assessment, and causal interpretation.

### 3.2. Patient Perspective

A formal written patient perspective was not obtained. During clinical evaluation, however, the patient repeatedly stated that his right hand moved on its own and that he was unable to control its spatial movements voluntarily. He described the right arm as if it consisted of separate parts loosely connected like a puppet. This description supports a subjective disturbance of agency, although no standardized agency or body-ownership assessment was performed.

## 4. Conclusions

Alien hand phenomena may rarely follow pontine hemorrhage and can overlap substantially with sensory ataxia, apraxia, and other post-stroke movement disorders. Diagnosis should be based on careful assessment of agency, limb ownership, autonomous apparently purposeful activity, intermanual conflict, grasping behavior, levitation, proprioception, and visuospatial control. Disruption of pontine proprioceptive and sensorimotor pathways may have contributed in this patient, but the mechanism remains hypothetical. When feasible, clinicians should consider EEG when events are paroxysmal, perform formal neuropsychological and functional assessments, document rehabilitation adherence and barriers, and record serial outcomes with standardized scales and video. Multicenter case registries and longitudinal studies are needed to clarify the spectrum, mechanisms, natural history, and rehabilitation response of infratentorial AHS.

## Figures and Tables

**Figure 1:**
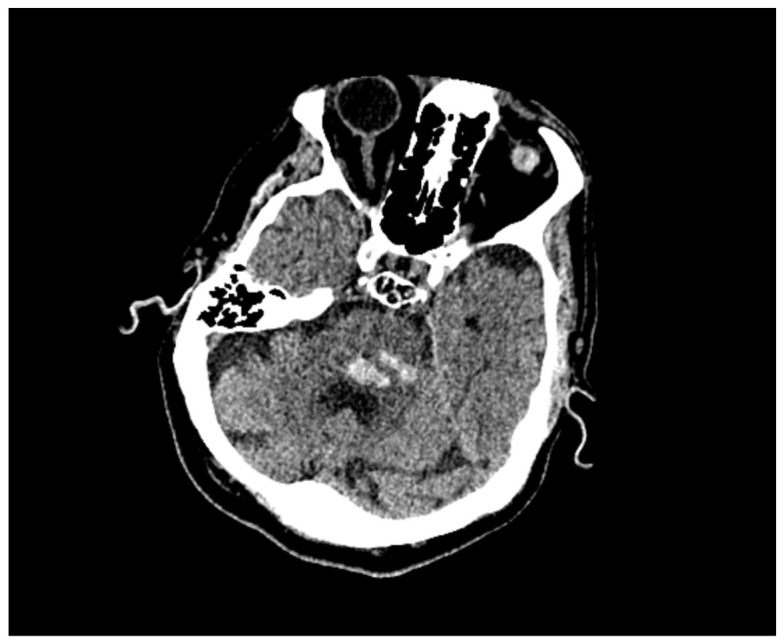
Non-contrast cranial CT demonstrating a left pontine hemorrhage.

**Figure 2:**
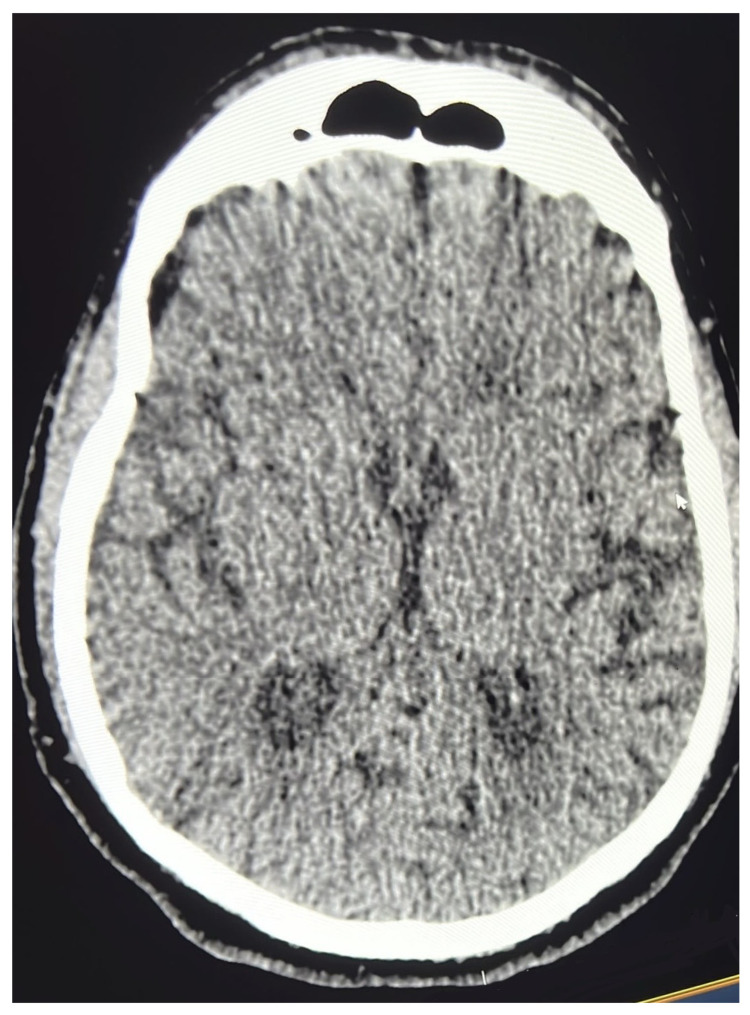
Representative axial non-contrast cranial CT image through the thalami, showing no evident thalamic hemorrhage or other gross structural lesion. A small acute ischemic lesion cannot be definitively excluded by CT alone.

**Table 1:** Timeline of the clinical course.

Time Point	Clinical Event
Day 0	Sudden sensation of heat in the head, transient loss of consciousness, fecal incontinence, fall, and facial injury.
Day 0	Cranial CT demonstrated a left pontine hemorrhage, while diffusion-weighted MRI showed no evidence of acute ischemia; the patient was admitted to the intensive care unit.
Days 1–14	Intensive care unit follow-up.
Week 2	Transfer to our hospital for neurological management.
Week 2	Right-sided involuntary hand movements, impaired spatial control, involuntary levitation, competitive reaching, proprioceptive impairment, and mild lower-limb involvement were documented.
Hospital stay	Serial cranial CT showed reduction in hemorrhage size. No specific pharmacological treatment was initiated. The NIHSS, assessed 15 days after admission to our hospital, was 6 points.
Discharge	Clinical condition was stable; cognitive-behavioral rehabilitation was recommended, but the patient was unable to participate because transportation difficulties prevented regular outpatient attendance; physical therapy and rehabilitation were recommended.
Approximately 3 months after discharge	According to information obtained from the patient’s physician relatives, abnormal movements had resolved and independent gait had improved. No formal neurological examination was performed.
Approximately 2 years after hemorrhage	According to information obtained from the patient’s physician relatives, no evident cognitive decline was reported. No formal neuropsychological assessment was performed.

**Table 2:** Differential diagnostic considerations.

Alternative Diagnosis	Reasoning in This Case
Sensory ataxia	Clearly present and likely contributory, but it did not fully account for loss of agency, involuntary levitation, competitive reaching, and autonomous apparently purposeful activity.
Chorea or dystonia	The movements were not primarily random, flowing, or continuously patterned. The thalamic-hand-like posture was noted but did not explain the full autonomous behavioral phenotype.
Ideomotor apraxia	Comprehension was preserved and the patient attempted commanded movements. Apraxia could not be formally excluded, but it would not alone explain involuntary competitive movements and the subjective loss of agency.
Functional neurological disorder	The syndrome emerged after a structural pontine hemorrhage with concordant focal sensory and ocular motor findings. No positive signs of inconsistency or incongruence were documented, although formal testing for functional signs was not performed.
Epileptic movements	EEG was not performed; however, the hand behavior was persistent rather than brief, stereotyped, or paroxysmal and occurred without altered awareness.
Drug-induced movements	No temporally related medication trigger was identified in the available records.

**Table 3:** Available follow-up and outcome information.

Time Point	Assessment Source	Clinical Status
Admission to our hospital	Direct neurological examination	Frequent involuntary right-hand behavior, levitation, impaired spatial control, intermanual conflict, inability to walk without support, and impaired sitting balance. The NIHSS, assessed 15 days after admission, was 6 points.
Discharge	Direct clinical observation	Clinically stable; physical therapy and rehabilitation recommended. No standardized functional score was recorded.
Approximately 3 months after discharge	Information from physician relatives	Abnormal movements reportedly resolved and independent gait improved. No formal neurological examination was performed.
Approximately 2 years after hemorrhage	Information from physician relatives	No evident cognitive decline was reported. No formal neuropsychological assessment was performed.

## Data Availability

The data supporting this report are available from the corresponding author upon reasonable request, subject to patient privacy and confidentiality.

## Abbreviations

AHS	Alien hand syndrome
CT	Computed tomography
EEG	Electroencephalography
ICD	International Classification of Diseases
ICF	International Classification of Functioning, Disability and Health
MRI	Magnetic resonance imaging
NIHSS	National Institutes of Health Stroke Scale
